# Psychological Capital and Innovative Work Behavior Among Pakistani Healthcare Professionals: Serial Mediation Through Career Adaptability and Work Engagement Based on the Career Construction Model of Adaptation

**DOI:** 10.3390/healthcare14152387

**Published:** 2026-08-04

**Authors:** Zane Asher Green, Haider Ali Shams, Murat Yıldırım, Farzana Ashraf, Feten Fekih-Romdhane, Juan Gómez-Salgado

**Affiliations:** 1 Contemporary Research Initiative, Preston University, Islamabad Campus, No. 85, Street 3, H-8/1, Islamabad 44000, Pakistan; zanearts@gmail.com; 2 Psychology Research Center, Khazar University, 41 Mahsati Ste., AZ1096 Baku, Azerbaijan; 3 Department of Computer Science, National University of Sciences and Technology, Kuch Road, Quetta 87300, Pakistan; 4 Department of Psychology, Faculty of Science and Letters, Ağrı İbrahim Çeçen University, 04100 Ağrı, Turkey; 5 Department of Humanities, COMSATS University Islamabad, Lahore Campus, Defence Road, Off Raiwind Road, Lahore 54000, Pakistan; 6 The Tunisian Center of Early Intervention in Psychosis, Department of Psychiatry “Ibn Omrane”, Razi Hospital, Manouba 2010, Tunisia; 7 Faculty of Medicine of Tunis, Tunis El Manar University, Tunis 1006, Tunisia; 8 Department of Sociology, Social Work and Public Health, Faculty of Labour Sciences, University of Huelva, 21071 Huelva, Spain; salgado@uhu.es; 9 Safety and Health Postgraduate Program, Universidad Espíritu Santo, Guayaquil 092301, Ecuador

**Keywords:** psychological capital, career adaptability, work engagement, innovative work behavior, healthcare practitioners, serial mediation

## Abstract

**Background:** This research explored how psychological capital (PsyCap) is associated with innovative work behavior (IWB) through the serial mediation of career adaptability (CA) and work engagement (WE) among Pakistani healthcare practitioners. The study was grounded in the Career Construction Model of Adaptation (CCMA). **Methods:** Data were collected from 355 healthcare practitioners. Model 6 of the PROCESS macro with bias-corrected bootstrapping was used to examine the proposed serial mediation model while controlling for the effects of gender, age, tenure, and professional category. **Results**: The total indirect effect through both serial mediators was statistically significant, suggesting that CA and WE help explain how PsyCap is associated with IWB. In line with the CCMA, the findings support a sequential pattern of associations whereby psychological resources (adaptive readiness) are associated with adaptable career functioning (adaptability resources), which in turn is linked with motivational engagement at work (adapting responses), and this state of engagement is subsequently associated with innovation-oriented actions (adaptation results). **Conclusions:** This study applies and extends the CCMA by examining its adaptation process in the healthcare sector, as well as conceptualizing WE as an adapting response and IWB as a behavioral adaptation outcome. The findings provide practical insights by highlighting the potential value of targeted interventions that strengthen PsyCap, CA, and WE to support IWB while also fostering the career adaptation process in healthcare organizations.

## 1. Introduction

Innovation is widely recognized as an important contributor to organizational success in the contemporary business landscape, which is driven by intense competition, rapid technological advancements, and a constant need for adaptation [1]. Employees are therefore highly encouraged to demonstrate innovative work behavior (IWB) within the organization [2]. This may include (a) finding new and better ways to perform their work, (b) identifying new technological advancements, (c) proposing ideas for new product or service applications or improving existing ones, and (d) finding novel production methods for upgrading/enhancing internal routines and production processes as well as advancing customer service excellence [3,4,5]. Overall, IWB can help organizations innovate and respond effectively to the volatile business environment through building and maintaining their competitive edge, productivity, and sustainability [6,7]. In the context of healthcare professionals, IWB has emerged as an important capability that may contribute to improving productivity. IWB can help healthcare organizations improve their operations and service delivery, as it permits practitioners to proactively seek and implement new ideas, processes, and solutions that may contribute to various aspects of patient care, organizational performance, productivity, and the overall healthcare system [8].

Pakistan’s healthcare system faces significant challenges in providing sustainable and equitable care [9,10]. Insufficient financing, outdated infrastructure, and a critical shortage of health professionals hinder the delivery of quality services, while a high burden of both communicable (e.g., tuberculosis and malaria) and non-communicable diseases (e.g., diabetes and cardiovascular disorders) strains the system further [10,11,12]. These issues combine to create significant healthcare disparities throughout the country [13]. Addressing these deeply rooted problems may require a strategic shift in focus; that is, healthcare institutions should prioritize cultivating IWB among their existing staff rather than relying exclusively on government funding. Moreover, technological investments and organizational restructuring as traditional elements influencing innovation may not be affordable solutions for many healthcare institutions [14]. Fostering innovation through solutions that tap into internal talent may therefore be a more feasible and cost-effective approach to developing more sustainable, people-centric healthcare delivery models [14], particularly in the context of a low- and middle-income country like Pakistan. This study proposes a mechanism for enhancing IWB among Pakistani healthcare professionals. Specifically, the study examines a serial mediation model grounded in the Career Construction Model of Adaptation (CCMA) [15]. In this model, psychological capital (PsyCap) is indirectly related to IWB through career adaptability (CA) and work engagement (WE). Consistent with the CCMA, the proposed relationships follow the career adaptation process, in which PsyCap, CA, WE, and IWB correspond to adaptive readiness, adaptability, adapting responses, and adaptation outcomes, respectively [15,16].

*PsyCap* represents a positive psychological state characterized by hope (perceiving pathways and having the motivation to achieve goals), optimism (positive expectations regarding present and future success), self-efficacy (confidence in one’s abilities), and resilience (bouncing back from adversity) as its four fundamental psychological resources [17]. Within the CCMA, these psychological resources are expressed through CA, which embodies psychosocial resources enabling individuals to navigate career transitions and manage the constant challenges of work by adjusting to new roles and developing resilience [16]. The effective use of the CA resources—concern (planning for the future), control (taking responsibility and action), curiosity (exploring options and information), and confidence (believing in one’s own abilities) [15]—may be reflected in WE (a positive, energizing state of vigor, dedication, and absorption) [18]. In turn, higher WE as a proximal adapting response may be associated with increased IWB (e.g., seeking opportunities, generating ideas, and implementing novel solutions) [19]. Hence, the hypothesized serial mediation model provides a theoretically grounded framework for understanding how PsyCap, CA, and WE may be reflected in IWB among healthcare professionals working in high-pressure environments.

### 1.1. Research Rationale and Significance

Considering the challenges associated with conducting longitudinal research among healthcare professionals, this study provides a useful first step toward understanding how career adaptation may be reflected through psychological resources, work-related states, and innovative behaviors in this occupational context. Moreover, by conceptualizing PsyCap as an indicator of adaptive readiness and IWB as an indicator of adaptation results, the study widens the range of constructs through which the CCMA may be conceptualized, examined, and applied. As a result, the findings provide a foundation for future longitudinal studies on the CCMA in healthcare settings.

This study addresses an important research gap in the literature by examining a serial mediation model (*PsyCap → CA → WE → IWB*) among Pakistani healthcare professionals, a context that has received limited attention. Previous research has demonstrated a direct link between PsyCap and IWB [20,21]. However, the precise mechanism through which PsyCap may contribute to IWB remains insufficiently understood. While one prior study demonstrated a serial mediation effect involving transformational leadership and WE among nurses in Iraq [22], the current study examines an alternative mechanism based on the sequential mediating roles of CA and WE. In doing so, the present study extends existing knowledge by providing a theoretically grounded explanation of how positive psychological resources may be reflected in IWB.

This research is relevant to the increasingly complex and demanding healthcare environment. In such environments, technical knowledge and professional competencies alone may not be sufficient to sustain effective performance. Instead, internal psychological resources such as PsyCap and CA may complement human capital by enabling practitioners to adjust and adapt to their work environment, remain engaged in their work, and perform their roles in more innovative ways.

In this research, PsyCap is conceptualized as an adaptive readiness resource within the CCMA framework. PsyCap reflects a malleable, state-like internal psychological capacity that integrates motivational, cognitive, and resilience-based capacities that may be developed and applied to cope with evolving work demands [23,24]. PsyCap is unlike broader personality-based constructs, such as core self-evaluations and proactive personality. Although these conceptualized have also been conceptualized as indicators of adaptive readiness, they represent relatively stable dispositional tendencies [25,26]. As a multi-layered personal resource, PsyCap, by contrast, represents a more active, flexible, and developable psychological resource system. From the perspective of Conservation of Resources Theory, PsyCap can operate as a dynamic resource reservoir that supports effective adaptation under demanding conditions [27,28], including those commonly encountered in healthcare settings. PsyCap is therefore conceptually consistent with the adaptive readiness component of the CCMA. Further, although WE has predominantly been examined as a positive organizational or adaptation outcome, only limited research has explicitly conceptualized WE as an adapting response within the CCMA framework [29]. Building on this perspective, this study conceptualizes WE as a proximal adapting response linking CA to IWB in healthcare contexts. Finally, by treating IWB as a distal adaptation outcome, the study broadens the CCMA’s adaptation outcome repertoire to include innovation-focused behaviors. Thus, the study extends the CCMA’s relevance for understanding adaptation processes and work-related behavioral outcomes in healthcare settings.

### 1.2. Theoretical Framework

This research contribution is rooted in the CCMA [15], which describes adaptation as a step-by-step process through which individuals construct their careers by managing career-related challenges, transitions, and evolving work demands. The CCMA proposes that adaptive readiness is reflected in adaptability resources, which are in turn expressed through adapting responses that ultimately support adaptation results [16,30]. Moreover, each component of the CCMA provides the conceptual basis for the next stage of adaptation. As a guiding process framework, the CCMA is used to structure and interpret the sequential relationships among psychological and behavioral variables in the proposed serial mediation model. The study demonstrates how the CCMA-based adaptation sequence may unfold within high-pressure, complex, and resource-constrained healthcare environments.

Healthcare practitioners possessing higher levels of PsyCap may approach these demanding work conditions with greater self-efficacy, optimism, hope (goal-directed thinking), and resilience [31]. These internal psychological strengths may be expressed through proactivity, flexibility, tenacity, and a future-oriented outlook that may help practitioners manage career-related responsibilities, challenges, and workplace changes. Such tendencies may correspond to higher levels of CA resources. Important for career self-management, these psychosocial resources include anticipating future work demands and proactively managing career-related tasks, confidently managing unfamiliar situations and challenges, exploring continuous learning and professional development opportunities, and adapting and adjusting to changing professional roles and expectations [15]. Stronger CA resources may be associated with higher levels of WE by helping healthcare practitioners remain actively involved in their work while sustaining emotional commitment and psychological connection to their professional roles. In healthcare settings, such adaptability is particularly important for adjusting to extensive workload, technological advancements, emotional strain, operational uncertainty, and evolving healthcare practices. These psychosocial competencies may subsequently be associated with higher WE as a proximal adapting response state characterized by vigor, dedication, and psychological investment in work roles [18]. Increased engagement may be accompanied by greater willingness to seek improved approaches for refining work methods, contribute constructive ideas for improving existing practices, and actively support innovation-based enhancements in healthcare service delivery and organizational functioning. As such, IWB may serve as an observable indicator of effective adaptation within high-pressure and high-stakes healthcare settings [5,19].

### 1.3. Hypothesis Development

#### 1.3.1. Psychological Capital → Innovative Work Behavior

PsyCap, representing a positive state of mind encompassing self-efficacy, hope, resilience, and optimism, is associated with IWB [20,21,32]. Employees with high PsyCap are more enthusiastic, energetic, and open to experiences, and as such, may demonstrate an increased willingness and competence to generate and embrace new ideas pertinent to enhancing their ability to innovate [33,34]. For instance, PsyCap has been shown to be associated with innovative behavior among nurses by serving as a psychological resource that may facilitate creative problem-solving and the application of ideas to address workplace challenges [35]. In addition, nurses with higher PsyCap are more likely to generate ideas, acquire sponsorship, and implement innovations as well as improve patient care [36]. PsyCap may be associated with motivational and psychological resources that may encourage employees to engage in innovative work behaviors in complex, dynamic, and demanding organizational settings [37]. In light of the above, we test the following hypothesis:

**H1.**  *PsyCap will be positively associated with IWB*.

#### 1.3.2. Psychological Capital → Career Adaptability

In the healthcare sector, PsyCap may strengthen employees’ capacity to cope with emotional and psychological demands (e.g., high stress levels, long hours, and exposure to difficult situations) and may help them explore new opportunities, take on new roles, and engage in continuous learning and skill development [38,39]. These career development competencies linked with PsyCap are, in essence, reflected in CA, which represents employees’ proactive readiness to perform their regular tasks, adapt to the dynamic job demands, and effectively address unexpected or challenging circumstances at work [29]. PsyCap reflects fundamental psychological strengths and motivation to manage career challenges and transitions. These may be associated with higher levels of CA [40]. Research has consistently demonstrated a positive association between PsyCap and CA [41,42,43]. Compared with human capital and social capital, PsyCap has also shown a stronger association with CA [44].

In line with the CCMA perspective, individuals with higher PsyCap are likely to be more adaptable; that is, they are better equipped to manage career demands and challenges. Furthermore, individuals who are confident and resilient are more likely to take initiative in their career development [45,46]. From the perspective of the CCMA, PsyCap may provide a foundation for CA because it may help individuals to embrace change with greater confidence, actively seek knowledge, develop relevant skills, and maintain a sense of purpose and direction in their careers [38,39]. Based on the empirical evidence and theoretical perspectives presented in this section, we test the following hypothesis:

**H2.**  *PsyCap will be positively associated with CA*.

#### 1.3.3. Career Adaptability → Work Engagement

CA has been associated with WE [47,48]. CA reflects psychosocial resources that may enable individuals to proactively plan their careers, feel responsible for their career development, remain open to exploring new opportunities, and gain confidence in their ability to navigate challenges [15]. As such, CA may be relevant for thriving in dynamic professional environments and has been linked with engagement at work. Furthermore, drawing on the CCMA logic, adaptability resources are linked to adapting responses [15]. CA may therefore be associated with WE, which reflects a proximal adapting response state characterized by vigor, dedication, and absorption in work roles [18]. Research has also indicated that CA is positively associated with WE [16,47,48,49]. Considering the above, we test the following hypothesis:

**H3.**  *CA will be positively associated with WE*.

#### 1.3.4. Work Engagement → Innovative Work Behavior

The CCMA also proposes that adapting responses are ultimately reflected in adaptation results [15]. In line with the CCMA, WE, in this study, is treated as an adapting response because it reflects an employee’s active motivational, cognitive, and emotional adjustment to evolving working conditions [15,50].

WE reflects an energetic and constructive work-related mindset that may contribute to positive organizational functioning [50,51]. IWB, by contrast, is conceptualized as an adaptation result primarily because it captures the observable behavioral outcome of successful adaptation in the workplace [16,30].

WE also reflects continuous mental and emotional involvement in work roles, where individuals remain actively engaged in performing their job tasks. During such periods, individuals may be more likely to demonstrate innovative behaviors aimed at addressing workplace challenges and improving operational practices [1]. Several studies have suggested a positive association between WE and IWB [6,52,53,54]. Hence, we test the following hypothesis:

**H4.**  *WE will be positively associated with IWB*.

#### 1.3.5. Career Adaptability and Work Engagement as Serial Mediators

Based on the central propositions of the CCMA as well as previous empirical evidence, this research proposes that CA and WE function as serial mediators through which PsyCap may be associated with IWB. CA refers to a set of psychosocial resources that help individuals manage career-related responsibilities, adapt to ever-evolving work demands, and respond constructively to changing professional contexts [15,16]. However, adaptability resources alone may not necessarily be expressed through IWB unless individuals remain psychologically invested and actively involved in their work roles.

In this context, WE captures a fulfilling and constructive work experience in which employees feel energetic, strongly dedicated to their work roles, and meaningfully absorbed in their daily tasks [18,50]. Higher engagement may be linked with greater willingness among employees to refine operational practices, address workplace challenges, and contribute to improvement-oriented initiatives that require innovative behaviors [1,55]. Previous research has reported positive associations among PsyCap, CA, WE, and IWB. These associations provide a reasonable basis for examining the proposed sequential pathway in this study [41,47,52,53]. Based on the literature and empirical evidence presented above, we test the following hypothesis:

**H5.**  *PsyCap will be indirectly associated with IWB through the serial mediation of CA and WE, while controlling for the effects of gender, age, tenure, and professional category*.

## 2. Method

This study was based on a cross-sectional survey design. It used self-report measures, which were administered to healthcare professionals in Pakistan. All procedures performed in the study were in accordance with the ethical principles outlined in the Declaration of Helsinki. Prior to data collection, this study was approved by the Ethical Review Board of COMSATS University Islamabad, Lahore Campus, vide Reference: HUM/CUI/LHR/2401. Participation in the study was voluntary.

### 2.1. Participants and Procedure

Three hundred and fifty-five healthcare professionals participated in the study. The sample comprised 194 (55%) men and 161 (45%) women recruited from 4 hospitals and 3 health centers situated in two major cities in Pakistan. The average age of the healthcare professionals was 34 years (*SD* = 9.69). The key sociodemographic characteristics of the study sample are presented in Table 1, including the different categories of healthcare employees who participated in the study. Eligibility criteria included being currently employed in a healthcare facility and voluntary participation in the study.

To explain the nature and scope of the study, the researchers formally contacted the appropriate departments (Human Resources and Research and Development), which facilitate data collection and research-related activities involving healthcare employees. After obtaining approval from each healthcare facility, each site was provided a set of questionnaires for completion by the different categories of its employees (see Table 1). The participants’ information sheet provided information about the purpose of the study and assured them of the anonymity and confidentiality of their responses. It also informed them that they could withdraw from participation at any stage without penalty. Before completing the questionnaire, each participant provided informed consent. To reduce social desirability concerns as well as encourage honest responses, participants completed the questions anonymously. The completed questionnaires were collected from each facility upon notification of their completion.

### 2.2. Instruments

A four-factor measurement model was tested, consisting of PsyCap, CA, WE, and IWB. PsyCap, CA, and WE were modeled based on their established dimensional structures. The questionnaires were administered in English. The medium of professional communication and documentation in Pakistani healthcare settings is English.

#### 2.2.1. The Compound Psychological Capital Scale (CPC-12)

The PsyCap of healthcare professionals was measured through the 12-item Compound Psychological Capital Scale (CPC-12) [56]. The scale assessed the four dimensions of PsyCap, namely hope (e.g., “I can think of many ways to reach my current goals”), optimism (e.g., “Overall, I expect more good things to happen to me than bad”), resilience (e.g., “When I’m in a difficult situation, I can usually find my way out of it”), and self-efficacy (e.g., “I can solve most problems if I invest the necessary effort”) on a 5-point Likert-type scale (1 = *strongly disagree*; 5 = *strongly agree*). This research used a total PsyCap score to represent a single overall psychological resource in line with previous research [57]. Lorenz et al. [56] reported an internal consistency reliability of 0.82. Based on the study sample, the scale demonstrated excellent internal consistency reliability (α = 0.95) and composite reliability (CR = 0.94). Additionally, it demonstrated adequate convergent validity (AVE = 0.56). Furthermore, the Heterotriat-Monotrait (HTMT) ratios between PsyCap and the other constructs ranged from 0.53 to 0.72. These were all below the recommended threshold of 0.85 and therefore supported discriminant validity. Higher scores on the scale indicate higher levels of PsyCap.

#### 2.2.2. Career Adapt-Abilities Scale–Short Form

This is a 12-item scale by Maggiori et al. [58], representing each of the four resources of CA through three items. The scale uses a 5-point Likert-type scale (1 = *not a strength*; 5 = *greatest strength*) for participants to rate each item. Sample items of the scale are “Becoming aware of the educational and vocational choices that I must make” (concern), “Counting on myself” (control), “Investigating options before making a choice” (curiosity), and “Learning new skills” (confidence). In this study, the global CA score was used to reflect a unified career adaptability resource consistent with prior research [59]. The same study reported a Cronbach’s alpha coefficient of 0.92 for the global scale [59]. In the study sample, the Cronbach’s alpha value was 0.96, composite reliability (CR) 0.95, and average variance extracted (AVE) 0.61. Further, the HTMT ratios between CA and the other constructs ranged from 0.53 to 0.82. The scale therefore supported discriminant validity. Higher scores on the scale indicate higher levels of CA.

#### 2.2.3. Utrecht Work Engagement Scale–9

This nine-item scale measures WE in terms of three dimensions, namely vigor (e.g., “At my job, I feel strong and vigorous”), dedication (e.g., “I am proud of the work that I do”), and absorption (e.g., “I am immersed in my work”) [60]. Each item of the scale is rated on a 5-point Likert scale (1 = *never*; 5 = *always*). In this study, the total sum of the nine items was taken to reflect overall WE. According to Tran et al. [61], the internal consistency reliability of the global scale amounted to 0.93. In this study, the scale demonstrated excellent internal consistency reliability (Cronbach’s α = 0.95) as well as composite reliability (CR = 0.95). Further, the scale demonstrated satisfactory convergent validity (AVE = 0.66). Additionally, HTMT ratios between work engagement and the other constructs ranged from 0.48 to 0.82. As such, the scale demonstrated adequate discriminant validity. Higher scores on the scale represent greater WE.

#### 2.2.4. Innovative Work Behavior

This nine-item scale, developed by Janssen [19], measures the degree to which employees demonstrate IWB. A stem—with what frequency do you engage in the behaviors listed below?—precedes the nine items. Sample items are: “Searching out new work methods, techniques or instruments” and “Transforming innovative ideas into useful applications.” Each item of the scale is rated on a 5-point Likert scale (1 = *never*; 5 = *always*). Janssen [19] reported an internal consistency reliability of 0.95 for the scale. An initial confirmatory factor analysis (CFA) of the original nine-item scale indicated that three items (Items 1, 5, and 6) did not perform adequately in the current sample. These items were therefore excluded. The revised six-item version was used in all subsequent analyses. The rationale for the revised scale and details of the CFA results are provided in the Preliminary Analyses section. Based on the study sample, the Cronbach’s α was 0.90 and CR 0.89. Further, the convergent validity was acceptable (AVE = 0.58). Also, the HTMT ratios between IWB and the remaining constructs ranged from 0.57 to 0.82. The scale therefore showed adequate discriminant validity. Higher scores on the scale suggest that employees demonstrate increased IWB.

### 2.3. Data Analyses

Preliminary analyses entailed computing descriptive statistics and intercorrelations among the study variables. Additionally, internal consistency reliability based on Cronbach’s alpha, CR, and AVE of each variable was determined as reported in the Instruments section. Also, HTMT ratios were calculated to determine the discriminant validity of each scale as presented in the Instruments section. All study variables were measured using self-report questionnaires completed by the same respondents at a single time point. Therefore, measures were taken to reduce the potential influence of common method bias (CMB). During data collection, participation was voluntary, responses were collected anonymously and treated confidentially, and participants were encouraged to answer honestly by emphasizing that no response was right or wrong. To further examine the potential influence of CMB, Harman’s single-factor test was conducted. The first factor accounted for 45.50% of the total variance, which was below the commonly recommended 50% threshold. This suggested that CMB was unlikely to pose a significant threat to the study findings [62,63].

To evaluate the adequacy of the four-factor measurement model, a CFA was conducted in LISREL 8.80 using maximum likelihood estimation. CFA results are reported in the Preliminary Analyses following Journal Article Reporting Standards (JARS) reporting standards. Model fit was assessed through multiple fit indices [64,65,66], including the comparative fit index (CFI; good fit ≥ 0.95; acceptable fit ≥ 0.90), non-normed fit index (NNFI; good fit ≥ 0.95; acceptable fit ≥ 0.90), incremental fit index (IFI; good fit ≥ 0.95; acceptable fit ≥ 0.90), standardized root mean square residual (SRMR; good fit ≤ 0.05; acceptable fit ≤ 0.08), root mean square error of approximation (RMSEA; good fit ≤ 0.06; acceptable fit ≤ 0.08), and normed Chi-Square (*χ*^2^/df; good fit < 3; acceptable fit < 5).

For the main analysis, Model 6 of the PROCESS macro (version 4.2) for SPSS 24 [67] was used to test the serial mediation model while controlling for gender, age, tenure, and professional category. A bias-corrected bootstrapping method with 10,000 samples was employed to estimate the indirect effects and their associated 95% confidence intervals (CI). This approach provided a robust basis for examining the sequential mediation pathway from PsyCap to IWB through CA and WE. Indirect effects were considered statistically significant when the 95% confidence interval did not include zero.

## 3. Results

### 3.1. Preliminary Analyses

The descriptive statistics as well as the correlations between the study variables are presented in Table 2. The skewness and kurtosis values were within the ±2 range and therefore indicated acceptable normality for ML estimation in CFA [68]. Additionally, the variables were positively correlated with each other. The correlations ranged between 0.43 and 0.72.

The study sample comprised healthcare professionals from a wide range of occupational groups. Therefore, a Kruskal–Wallis test was performed to determine whether IWB varied by profession. Results indicated that IWB differed significantly among professional categories, *χ*^2^ (13) = 35.87, *p* = 0.001. As a result, professional category was added as an additional covariate in the serial mediation analysis.

Janssen’s [19] original nine-item Innovative Work Behavior scale was subjected to an initial CFA. The initial model did not satisfactorily fit the data, *χ*^2^ (23) = 193.58, *p* < 0.001; *χ*^2^/*df* = 8.42; RMSEA = 0.15; RMSEA 90% CI [0.13–0.17]; SRMR = 0.11; CFI = 0.92; NNFI = 0.87; IFI = 0.92. The standardized factor loadings showed that three items—“Creating new ideas for difficult issues” (Item 1), “Acquiring approval for innovative ideas” (Item 5), and “Making important company members enthusiastic for innovative ideas” (Item 6)—performed poorly, with loadings of 0.02, 0.10, and 0.06, respectively. Hence, these items were excluded from the measurement model.

A possible explanation for the poor performance of the three items lies in the diversity of the healthcare sample. Item 1 refers to creating new ideas for *difficult issues*, which may have been interpreted as addressing particularly complex or daunting problems that many healthcare employees were not expected to resolve routinely because of their designation or professional responsibilities. By contrast, identifying improved work methods or coming up with practical solutions may be behaviors that are more relevant to a wide range of healthcare occupations. In a similar vein, Items 5 and 6 focus on obtaining approval for innovative ideas and generating enthusiasm among influential organizational members. These behaviors may depend on employees’ organizational position, level of authority, and opportunities to influence decision-making. As such, these behaviors were likely to vary among the different professional groups in the study sample. The remaining six items in the IWB scale, however, reflected innovation-related behaviors that were more uniformly applicable to the different healthcare professionals represented in the sample. These included more generalized behaviors such as identifying improved work methods, generating practical solutions, supporting innovation, implementing new ideas, and evaluating their usefulness.

A second CFA of the revised six-item IWB scale demonstrated a satisfactory fit to the data, *χ*^2^ (5) = 12.90, *p* < 0.001; *χ*^2^/*df* = 2.58; RMSEA = 0.067; RMSEA 90% CI [0.022–0.11]; SRMR = 0.016; CFI = 0.99; NNFI = 0.98; IFI = 0.99. The six items showed satisfactory standardized factor loadings ranging from 0.63 to 0.86. This six-item scale was subsequently incorporated into the overall four-factor measurement model. Future research should, however, examine the revised six-item version of the scale using independent healthcare samples to confirm its measurement properties.

Results of CFA indicated that the hypothesized four-factor model (PsyCap, CA, WE, and IWB) demonstrated a generally acceptable fit to the study data, *χ*^2^ (691) = 2601.93, *p* < 0.001; *χ*^2^/*df* = 3.77; RMSEA = 0.088; RMSEA 90% CI [0.084–0.091]; SRMR = 0.051; CFI = 0.97; NNFI = 0.96; IFI = 0.97. The incremental and absolute fit indices were adequate, and the normed Chi-Square and SRMR were within acceptable limits. The RMSEA was slightly above the recommended 0.08 threshold. However, the remaining fit indices supported the adequacy of the measurement model, particularly when considered in light of the model complexity, sample size, and degrees of freedom. This finding is in line with prior evidence that RMSEA is sensitive to model complexity and degrees of freedom (Chen et al., 2008; Kenny et al., 2015; Marsh et al., 2004) [69,70,71]. Considering the full set of indices, the measurement model appears to represent the study data satisfactorily. This lends support to the construct validity of the study measures and provides sufficient confidence to proceed with the subsequent serial mediation analysis. The model was refined by using only theoretically justified post hoc modifications. This included item deletion as well as the estimation of selected error covariances in line with established guidelines for improving theory-driven model fit [64,65,72]. In addition, error covariances were estimated between CA4-CA5, WE1-WE6, and IWB7-IWB8, IWB7-IWB9, and IWB8-IWB9 based on theoretical considerations as well as item similarity. As a result of these refinements, an acceptable-fitting measurement model that was consistent with theory was obtained. The convergent validity was supported, as all retained items loaded significantly on their respective constructs. The preliminary analyses, therefore, provided sufficient support for proceeding with the serial mediation analysis.

### 3.2. Serial Mediation Analysis

**Figure 1:**
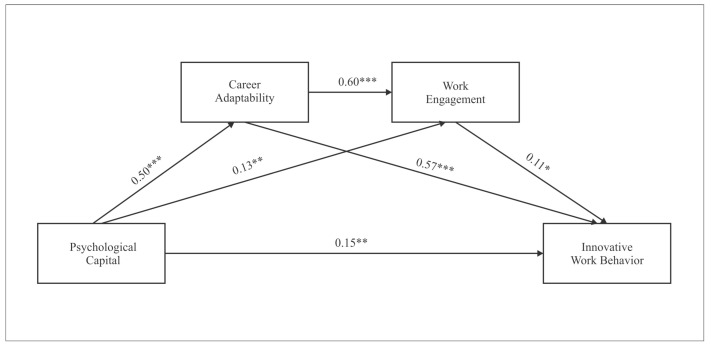
The serial mediation model. Note: * *p* < 0.05, ** *p* < 0.01, *** *p* < 0.001.

The serial mediation analysis indicated significant indirect effects based on the bootstrap sampling procedure. PsyCap represented the independent variable (X), CA as the first mediator (M1), WE as the second mediator (M2), and IWB as the dependent variable (Y). Figure 1 depicts the results of the mediation model that examines the role of CA and WE as serial mediators in the relationship between PsyCap and IWB, while controlling for the effects of gender, age, tenure, and professional category.

Results presented in Table 3, Table 4, and Figure 1 indicated significant associations between the study variables. PsyCap was directly associated with IWB (*B* = 0.08; *SE* = 0.02; *t* = 3.44; *p* < 0.01) as shown in Table 3. This supported Hypothesis 1. As regards the covariates, gender (*B* = −0.18; *SE* = 0.38; *t* = −0.47; *p* = 0.64), age (*B* = 0.03; *SE* = 0.05; *t* = 0.54; *p* = 0.59), tenure (*B* = −0.03; *SE* = 0.08; *t* = −0.41; *p* = 0.68), and professional category (*B* = 0.05; *SE* = 0.05; *t* = 0.89; *p* = 0.37) had no significant effect on IWB.

Furthermore, as per Table 3, PsyCap was positively associated with CA (*B* = 0.51; *SE* = 0.05; *t* = 10.82; *p* < 0.001), supporting Hypothesis 2. With respect to the covariates, gender (*B* = −0.67; *SE* = 0.90; *t* = −0.75; *p* = 0.45), age (*B* = −0.12; *SE* = 0.11; *t* = −1.08; *p* = 0.28), tenure (*B* = 0.26; *SE* = 0.19; *t* = 1.35; *p* = 0.18), and professional category (*B* = −0.09; *SE* = 0.12; *t* = −0.74; *p* = 0.46) had no significant influence on CA.

In addition, as presented in Table 3, CA demonstrated a positive association with WE (*B* = 0.48; *SE* = 0.04; *t* = 13.05; *p* < 0.001), confirming Hypothesis 3. Relating to the covariates, gender (*B* = 0.46; *SE* = 0.62; *t* = 0.75; *p* = 0.46), age (*B* = 0.05; *SE* = 0.08; *t* = 0.70; *p* = 0.48), tenure (*B* = −0.07; *SE* = 0.13; *t* = −0.51; *p* = 0.61), and professional category (*B* = 0.001; *SE* = 0.09; *t* = 0.01; *p* = 0.99) had no significant influence on WE.

Also, as shown in Table 3, WE was positively associated with IWB (*B* = 0.07; *SE* = 0.03; *t* = 2.28; *p* < 0.05). This finding supported Hypothesis 4. Concerning the covariates, gender (*B* = −0.18; *SE* = 0.38; *t* = −0.47; *p* = 0.64), age (*B* = 0.03; *SE* = 0.05; *t* = 0.54; *p* = 0.59), tenure (*B* = −0.03; *SE* = 0.08; *t* = −0.41; *p* = 0.68), and professional category (*B* = 0.05; *SE* = 0.05; *t* = 0.89; *p* = 0.37) had no significant effect on IWB.

Moreover, as shown in Table 4, the indirect effect of PsyCap on IWB via CA (X → M1 → Y) was significant (*B* = 0.16; Boot*SE* = 0.02; 95% bootstrap CI [0.1088, 0.2072]). Similarly, the indirect effect of PsyCap on IWB via WE (X → M2 → Y) was also significant (*B* = 0.01; Boot*SE* = 0.00; 95% bootstrap CI [0.0007, 0.0193]). As a consequence of the serial mediating effect, the indirect effect of PsyCap on IWB via both CA and WE (X → M1 → M2 → Y) was significant (*B* = 0.02; Boot*SE* = 0.01; 95% bootstrap CI [0.0026, 0.0381]), while controlling for the effects of gender, age, tenure, and professional category. WE contributed to the indirect association between PsyCap and IWB. Its contribution, however, was more modest than that of CA. PsyCap, CA, and WE collectively accounted for 54.40% of the variance in the dependent variable, IWB (*R*^2^ = 0.54). The above results confirmed Hypothesis 5.

## 4. Discussion

The serial mediation model in this study provides a valuable framework for understanding how psychological resources may be strategically used to support IWB in a dynamically changing and demanding healthcare environment. Rather than viewing these associations in isolation, the findings suggest that these associations may be better understood as interconnected stages of the adaptation process proposed by the CCMA.

Results revealed that PsyCap was related to IWB. Prior research has also suggested a relationship between PsyCap and IWB [20,21,32]. In the context of healthcare, PsyCap may be associated with an innovative mindset that supports practitioners in identifying and implementing improvements to patient care, clinical practices, workflows, and service delivery [35,36]. Rather than suggesting a direct effect, these findings suggest that PsyCap may serve as a vital psychological resource associated with innovation-related behaviors among healthcare professionals working in demanding organizational settings.

In addition, results indicated that PsyCap was related to CA. Previous research has also suggested the same [41,73]. These findings suggest that PsyCap may represent an important psychological resource associated with higher levels of CA among healthcare professionals. This may be important within healthcare settings because healthcare professionals routinely face changing clinical practices, emerging technologies, and continually shifting patient needs. Under these conditions, hope, optimism, resilience, and self-efficacy may provide psychological resources associated with the development and application of adaptability resources when responding to the challenges of everyday professional practice.

Results also indicated a relationship between CA and WE. Previous scholarly work has also shown a relationship between CA and WE [16,35,48,49]. Career adaptability embodies psychosocial resources that may be associated with proactive career management, continuous professional development, adjustment to changing work demands, effective management of uncertainty, and recovery from setbacks [15,16,74]. In line with the CCMA, this finding suggests that career adaptability may be considered more than a desirable career-related attribute. It may instead be viewed as a psychosocial resource through which employees’ psychological strengths may find expression in the motivational engagement they bring to their everyday work. This may be particularly relevant in complex, challenging, and unpredictable healthcare settings, where practitioners are often required to make continuous adjustments to evolving demands and responsibilities.

Furthermore, results indicated a relationship between WE and IWB. This relationship has also been supported by prior studies [6,52,53,54]. The present findings extend this pattern to Pakistani healthcare professionals. Highly engaged hospital employees may invest greater cognitive, emotional, and behavioral energy in their work, which may be associated with greater willingness to consider innovative approaches to clinical practice and service delivery [6,52,53,54]. This may be relevant in dynamic healthcare environments, where practitioners are frequently required to balance efficiency, quality care, and continuous adaptation [10,11,12]. In complex and demanding healthcare settings, highly engaged employees are likely to invest greater energy and attention in their work. This may encourage them not only to respond to immediate work demands but also to identify opportunities for refining existing practices and enhancing the quality of service delivery.

The indirect effect of PsyCap on IWB through the serial mediation of CA and WE—while controlling for the effects of gender, age, tenure, and professional category—was significant. In the demanding healthcare environment, practitioners with strong PsyCap are more confident in their ability to solve problems, remain hopeful about their career future, and are better able to recover from daily stressors and setbacks [56], such as high patient acuity or staff shortages. The observed association between PsyCap and CA is consistent with the view that positive psychological resources may coincide with greater readiness to adapt to evolving professional responsibilities, changing healthcare policies, technological developments, and dynamic patient needs [38,39,40]. Similarly, the association between CA and WE suggests that adaptability resources may coincide with greater psychological investment in work, reflected through vigor, dedication, and absorption [49,60,75,76,77]. The association between WE and IWB further suggests that healthcare professionals who remain psychologically invested in their work may also be more inclined to generate, champion, and implement innovative ideas within their work roles [1]. Viewed collectively, these interpretations suggest that innovation in healthcare may be linked not only to employees’ psychological strengths but also to how those strengths are embodied in career adaptability and sustained engagement in their daily work.

### 4.1. Theoretical Contribution

To the best of our knowledge, this research is among the few to apply the CCMA within the healthcare setting. In this regard, it advances the CCMA in different ways and makes important contributions to vocational, positive, and organizational psychology literature. First, this study provides a clear understanding of how PsyCap is associated with IWB through a sequential mechanism in which CA and WE function as intermediary processes in healthcare settings. CCMA highlights CA as a vital mechanism for managing career demands, complex problems, and uncertain conditions. However, research has yet to clarify how psychological resources may contribute to the development of career adaptability and how it may, in turn, be linked with motivational states and behavioral outcomes at work within a single explanatory framework. This study addresses this gap by examining associations in line with the CCMA-based sequential pathway linking PsyCap, CA, WE, and IWB. Instead of examining these constructs as isolated associations, the study considers them as conceptually connected stages of the career adaptation process. This study uses context-relevant psychological and behavioral constructs in healthcare settings rather than a purely conceptual mapping of the core components of the CCMA. In doing so, the study demonstrates how the adaptation sequence proposed by the CCMA may be operationalized through occupation-relevant psychological resources, motivational states, and work behaviors that reflect the realities of healthcare practice. This extends previous applications of the CCMA by illustrating how its adaptation sequence may be examined using context-specific constructs that are directly relevant to healthcare professionals. As such, the study demonstrates the applicability of the CCMA as a process-oriented framework to explain how psychological resources may be associated with motivational experiences that are ultimately linked with innovative workplace behavior.

Second, this research explains how PsyCap, as a foundational resource, may shape CA. Rather than assuming that PsyCap and CA work independently with no connection, the findings suggest that higher PsyCap is associated with higher CA. This suggests that CA is linked with the availability of psychological resources that individuals already possess. As such the findings suggest that career adaptability may be understood not only as an enduring career-related capacity but also one that is associated with developable psychological resources. In terms of the CCMA, the findings support the idea that adaptive readiness is not merely a passive antecedent, but may be meaningfully associated with activating and reinforcing adaptability resources in real work settings. This extends the conceptualization of adaptive readiness within the CCMA by illustrating how it may be represented through a developable psychological resource rather than solely through relatively stable personal characteristic. Consequently, the findings broaden the ways in which adaptive readiness may be operationalized and examined in future CCMA research.

Third, this contribution conceptualizes CA as an immediate process linking personal resources to work outcomes. Rather than treating adaptability as an outcome-oriented competence, the findings show that CA may be associated with employees’ motivational experiences at work. This suggests that adaptable career functioning is not merely a career-related capability, but may also be linked to sustained psychological engagement at work. As such, the CCMA goes beyond explaining career processes by also accounting for work-related motivational functioning. This extends the application of the CCMA by illustrating how career adaptability may connect adaptive readiness with employees’ day-to-day motivational experience in occupational settings. From this perspective, WE may be understood as a work-related motivational state through which adaptability resources become associated with employees’ workplace behavior.

Fourth, by demonstrating that WE is associated with IWB, the study broadens the explanatory scope of CCMA, which has largely been used to explain adaptation, adjustment, and well-being outcomes. According to the findings, the model may also provide valuable insights into how adaptable and engaged employees may contribute to innovation-oriented behaviors at work. In doing so, the study extends the CCMA by illustrating that the adaptation process may also be understood in relation to workplace behaviors that contribute to organizational functioning, rather than being limited to individual career or well-being outcomes. As a result, the findings broaden the range of adaptation outcomes through which the CCMA may be applied and examined in future research.

Finally, by integrating PsyCap, CA, WE, and IWB into a single sequential framework, the study provides a structured explanation of how psychological resources are associated with employees’ ability to adapt to career-related demands, which is then linked with greater motivational engagement at work. This psychological engagement is subsequently associated with innovation-related workplace behavior. This study positions these relationships as conceptually connected components of a single adaptation sequence that aligns with the CCMA. Because this sequence follows the conceptual ordering proposed by the CCMA, it provides a theoretically coherent explanation of how psychological resources may be associated with innovation through successive adaptation processes. This perspective differs from treating these constructs as independent or interchangeable workplace variables. Instead of focusing on individual relationships alone, the study provides a clearer understanding of how PsyCap, CA, WE, and IWB may fit together as successive stages of the adaptation process proposed by the CCMA. This integrated perspective provides a clearer understanding of how career adaptability may be reflected through psychological resources, motivational experiences, and innovation-related workplace behavior in healthcare settings.

Overall, the study extends the application of the CCMA by demonstrating that its adaptation process can be meaningfully examined through healthcare-relevant psychological resources, work-related motivational states, and innovation-related behaviors. Collectively, the findings illustrate the potential of the CCMA as a process-oriented framework for examining career adaptation in occupational contexts characterized by continuous change, uncertainty, and demanding work conditions. The proposed adaptation sequence may therefore be relevant in occupations characterized by high adaptation demands. However, its applicability to more stable occupational contexts remains to be established.

### 4.2. Practical Implications

The serial mediation model may help human resource departments and healthcare administrators to gain insights into the distinct psychological mechanisms through which employees’ inherent strengths and capacities (PsyCap) may ultimately be reflected in innovative behaviors. The model provides a structured framework for designing targeted interventions and strategies. However given the cross-sectional nature of this study, these implications should be viewed as theoretically informed possibilities rather than evidence of intervention effectiveness. Longitudinal and experimental is needed to determine whether interventions targeting PsyCap, CA, and WE produce the outcomes in healthcare settings. Instead of generalized capacity-building initiatives, human resource departments (HRDs) may implement programs that build PsyCap, foster CA, and boost WE. Such initiatives may create conditions that are more conducive to organizational innovation and may also support talent management and employee retention.

PsyCap interventions may be designed to equip healthcare practitioners with the core resources to navigate and effectively manage the emotional and psychological demands inherent in the modern healthcare environment. This positive psychological state may serve as an important psychological resource associated with the sequential process examined in this study. PsyCap development interventions rooted in psychological capital research stress that hope, optimism, resilience, and efficacy can be intentionally developed through structured intervention strategies [78,79]. In addition, healthcare organizations and their administration can take simple steps to foster PsyCap among healthcare practitioners, including: (a) fostering a positive culture that offers reasonable autonomy and manageable workloads, as supported by the Job Demands–Resources Model [80], (b) training department managers and team leaders to provide coaching and model positive behaviors [24], (c) providing training in psychological resilience and stress management, which is widely supported in occupational health psychology literature [81], and (d) developing informal peer support networks to improve coping capacities in high-stress healthcare organizations [82]. Furthermore, HRDs may need to refine their recruitment and selection criteria. They may benefit from considering PsyCap-related qualities during the recruitment and selection process, as individuals with higher PsyCap have been associated with favorable outcomes, such as increased engagement and performance [27].

Interventions designed to strengthen CA may focus on enhancing specific skills related to its four dimensions—concern, control, curiosity, and confidence (e.g., motivating employees to be forward-looking and prepare for the future, take charge of their professional lives, explore different opportunities, be receptive to new experiences, maintain professional relevance, invest in personal growth, and strengthen self-efficacy to solve problems and overcome obstacles)—derived from the conceptualization of the construct by Savickas and Porfeli (2012) [15]. Although these strategies are conceptually consistent with the current findings as well as exiting literature, their effectiveness in producing the outcomes examined in this study requires confirmation through future intervention-based or longitudinal research. Other strategies for enhancing CA among healthcare professionals may include: (a) offering mentorship opportunities and support for active participation in online platforms and professional organizations to build connections with peers and leading medical practitioners in the field; (b) creating opportunities for aspiring leaders to lead projects to test their ability to handle responsibility and build their confidence; (c) encouraging employees to discuss their aims and aspirations with their medical supervisors and HRDs; (d) providing resources for skill-building to help employees navigate uncertainty and embrace new opportunities for innovation; (e) fostering continuous learning through mobile apps for *on-the-go learning*, webinars, VR/simulations for risk-free practice, and blended learning programs; (f) creating opportunities for employees to work in different departments, roles, or settings to gain a broader perspective, and (g) developing employee skills in such areas as communication, empathy, and resilience to enable them to effectively adapt to healthcare challenges [83,84,85,86].

Hospital administration, department managers, medical supervisors, and human resource teams may need to create an environment that is conducive to higher levels of WE by providing employees with access to organizational resources and support needed to remain psychologically invested in their work. These recommendations should be interpreted as theoretically informed implications derived from the observed associations rather than as evidence of causal intervention effects. Strategies aimed at enhancing WE are well-supported by the Job Demands–Resources Model (Bakker and Demerouti, 2007) [80]. Major strategies in this regard as applied to the healthcare sector include: (a) recognizing healthcare workers’ contributions to maintain motivation and focus on innovative tasks, (b) establishing clear and open lines of communication to ensure that all employees are informed and valued and are empowered to share their insights and feedback, (c) ensuring that healthcare employees experience their work as meaningful, (d) providing adequate job resources (e.g., autonomy, professional development opportunities, and supportive relationships) and promoting a healthy work–life balance to reduce stress and burnout, (e) offering diversity and inclusion training to promote understanding and respect for different cultural backgrounds because an inclusive workplace culture contributes to a collaborative and innovative workplace atmosphere, and (f) offering resources and support for mental well-being because of the stressful and traumatic situations routinely encountered by many healthcare workers [87,88,89].

## 5. Limitation and Future Research

This contribution has three major limitations that can be addressed through future research. Firstly, the measures assessing PsyCap, CA, WE, and IWB may have introduced response biases on account of social desirability and the use of a single method for data collection (CMB). Steps were taken to minimize CMB during data collection, and Harman’s single-factor test suggested that it was unlikely to be a major concern. Nevertheless, the findings should be interpreted with caution because all variables were measured using self-report questionnaires completed by the same respondents at a single time point. Also, due to the lack of full self-insight, participants may not have been able to provide accurate self-ratings on the four measures. Future research may therefore consider multiple methods of data collection. For instance, peer evaluations, supervisor ratings, or where appropriate objective indicators may also be incorporated. Also, a future study in which a rich qualitative component supports the quantitative results may make meaningful contributions to the existing literature.

Secondly, because of the cross-sectional survey design, it may not be possible to conclusively demonstrate the serial mediation path from PsyCap to IWB. As such, alternate causal paths may exist, including reverse causation. In the future, temporal precedence among the variables may be ascertained by systematically collecting data for each variable at different time points. This is likely to provide stronger evidence for the hypothesized serial mediation path as well as the absence of reverse causation. However, the proposed mediation model remains theoretically meaningful because it is rooted in the CCMA. Although the cross-sectional design limits conclusions about the temporal ordering of the variables, the CCMA provides a well-established process framework for meaningfully interpreting the observed pattern of associations. Thus, the proposed serial mediation model should be interpreted as a theoretically informed explanation of the observed associations rather than definitive evidence of the underlying adaptation process.

Thirdly, as the findings of the serial mediation model are specific to healthcare professionals in Pakistan, and were obtained from a heterogeneous sample of healthcare-related professionals working in different healthcare institutions, future studies may focus on enhancing the generalizability and external validity. The revised six-item IWB scale demonstrated satisfactory psychometric properties in the present sample. Future research should cross-validate this shortened version in independent healthcare samples to determine whether its psychometric properties and measurement structure can be replicated across different healthcare settings. The present study also included healthcare professionals from a broad range of clinical and non-clinical occupational groups. While this occupational diversity enhances the ecological relevance of the findings, it may have influenced both the expression and measurement of IWB and may also be associated with occupation-specific differences that were not explicitly modeled. These potential differences should therefore be considered when interpreting the findings and assessing their generalizability. Future studies may investigate more homogeneous professional groups and compare occupational categories to determine whether the observed relationships differ across healthcare professions. In this regard, the serial mediation model may be applied to healthcare professionals in other countries as well as to other service sectors (e.g., education, financial services, hospitality, and entertainment) in both Pakistan and abroad. Future studies may also examine whether the proposed relationships remain consistent among different healthcare professions and organizational settings. Such research would deepen our understanding of how the adaptation process proposed by the CCMA may be reflected differently from one occupational and cultural context to another. Moreover, such research would also help identify the organizational and occupational conditions under which the proposed adaptation sequence is more or less likely to be observed.

Additionally, there could be several research prospects stemming from the current mediation model. Possible boundary conditions that might influence the strength of the proposed relationships could be tested. For instance, the degree of job autonomy might strengthen or weaken the influence of PsyCap and WE on IWB. Also, organizational support or organizational climate might influence the impact of personal resources (PsyCap and CA) on IWB. Future research may also investigate other serial mediators (e.g., perceived organizational support and psychological safety, CA and creative self-efficacy, CA and occupational self-efficacy, and WE and work well-being) between psychological resources and IWB, as well as other relevant outcomes, such as job performance, work well-being, job satisfaction, or organizational commitment.

## 6. Conclusions

Based on the CCMA framework, the study concludes that irrespective of gender, age, tenure, and professional category, PsyCap may be associated with higher IWB among healthcare professionals in Pakistan, not only directly but also indirectly through the serial mediation of CA and WE. In essence, these findings demonstrate a sequential mechanism in line with the CCMA whereby psychological resources are associated with adaptable career functioning, which is linked with motivational engagement at work, and this engaged state is ultimately associated with innovation-related workplace conduct. Considered collectively, the findings suggest that the adaptation process proposed by the CCMA may be reflected through healthcare practitioners’ psychological resources, career adaptability as a psychosocial resource, motivational experiences at work, and innovation-related behaviors. This provides a meaningful illustration of how career adaptation may be expressed in everyday realities of healthcare practice.

The findings suggest that PsyCap is an important personal resource that may initiate the process of healthcare innovation. Higher levels of PsyCap may enhance healthcare workers’ capacity to adapt to career changes and challenges. Such adaptable career functioning (CA), in turn, may serve as an important mechanism for strengthening healthcare employees’ WE. Finally, this motivational engagement may be associated with greater IWB. Engaged practitioners are more likely to identify, advocate, and apply novel ideas, perspectives, and/or processes. These findings collectively suggest that fostering positive psychological resources among healthcare practitioners may present a promising avenue for supporting a more adaptable, engaged, and innovative workforce. However, because this study is cross-sectional and was conducted within Pakistani healthcare institutions, these implications should be considered as theoretically informed possibilities that require confirmation through longitudinal and intervention-based research before firm practical recommendations can be made.

## Figures and Tables

**Table 1:** Key sociodemographic characteristics of the sample (N = 355).

Variable	Frequency	%
Gender		
Male	194	55
Female	161	45
Age *M* = 34 years; *SD* = 9.69		
Tenure *M* = 7.40 years; *SD* = 5.37		
Healthcare Professionals		
Physicians	36	10
Mental Health Professionals	12	3
Medical Assistants	43	12
Nurses	38	11
Therapists	18	5
Healthcare/Nursing Assistants	48	14
Laboratory Staff	17	5
Radiology Staff	10	3
Emergency Department Staff	26	7
Administrative Staff	35	10
Pharmacy Staff	17	5
Managers	24	7
Receptionists and Patient Enrollment Staff	19	5
IT Staff	12	3

**Table 2:** Descriptive statistics, internal consistency reliability, and correlations.

No.	Variable	*M*	*SD*	Skew	Kurt	1.	2.	3.	4.
1.	PsyCap	43.65	9.48	−0.38	−0.29	—	0.50	0.43	0.48
2.	CA	44.72	9.55	−0.36	−0.13		—	0.66	0.72
3.	WE	32.39	7.69	−0.25	−0.38			—	0.55
4.	IWB	20.69	5.14	−0.08	−0.59				—

Note. All correlations are significant at *p* < 0.001.

**Table 3:** Standardized and unstandardized direct estimates.

Predictor		Outcome	*B*	*β*	*S.E.*	*p*
PsyCap	→	CA	0.51	0.50	0.05	<0.001
CA	→	WE	0.48	0.60	0.04	<0.001
PsyCap	→	WE	0.10	0.13	0.04	<0.01
WE	→	IWB	0.07	0.11	0.03	<0.05
PsyCap	→	IWB	0.08	0.15	0.02	<0.01
CA	→	IWB	0.31	0.57	0.03	<0.001

**Table 4:** Unstandardized indirect effects.

Predictor		Mediator		Outcome	*B*	*SE*	LLCI	ULCI
Total					0.18	0.02	0.1417	0.2294
PsyCap	→	CA	→	IWB	0.16	0.02	0.1088	0.2072
PsyCap	→	WE	→	IWB	0.01	0.00	0.0007	0.0193
PsyCap	→	CA → WE	→	IWB	0.02	0.01	0.0026	0.0381

## Data Availability

The data presented in this study are available on request from the corresponding author due to confidentiality considerations and the privacy of the participants.
